# Sexual size dimorphism in musteloids: An anomalous allometric pattern is explained by feeding ecology

**DOI:** 10.1002/ece3.2480

**Published:** 2016-10-29

**Authors:** Michael J. Noonan, Paul J. Johnson, Andrew C. Kitchener, Lauren A. Harrington, Chris Newman, David W. Macdonald

**Affiliations:** ^1^ Wildlife Conservation Research UnitZoology DepartmentThe Recanati‐Kaplan CentreUniversity of OxfordTubneyAbingdonUK; ^2^ Department of Natural SciencesNational Museums ScotlandEdinburghUK; ^3^ Institute of GeographySchool of GeosciencesUniversity of EdinburghEdinburghUK

**Keywords:** allometry, diet, Musteloidea, Rensch, resource dispersion, sociality, SSD

## Abstract

Rensch's rule states that sexual size dimorphism (SSD) increases with body size in taxa where males are larger, and decreases when females are larger. The dominant explanation for the trend is currently that competitive advantage for males is greater in larger individuals, whereas female size is constrained by the energetics of rearing offspring. This rule holds for a variety of vertebrate taxa, and opposing trends are rare. We examine the allometry of SSD within the Musteloidea and demonstrate a hypo‐allometry contrary to Rensch's rule, with lower SSD associated with larger body size. We provide evidence that feeding ecology is involved. Where diet promotes group‐living, the optimal strategy for the males of larger species is often not to attempt to defend access to multiple females, obviating any competitive advantage of relatively greater size. We conclude that the effect of feeding ecology on mating systems may be a hitherto neglected factor explaining variation in SSD.

## 1 Introduction

Dimorphism in secondary sexual characteristics—those not directly involved with the reproductive process—of sexually reproducing species has long attracted the attention of biologists. Darwin (1871) was among the first to survey the diversity of sexual dimorphism across the animal kingdom and to speculate on its causes. One of the most conspicuous dimorphisms is where the sexes differ in size (sexual size dimorphism; henceforth SSD), which arises when the rate of selection for body size in one sex exceeds that in the other. Three main hypotheses explaining SSD have been proposed (Hedrick & Temeles, 1989): (1) sexual selection, where males compete for access to mates, or females preferentially select larger males; (2) food competition, where dimorphism reduces competition between sexes; and (3) differences in reproductive strategies between sexes driving selection for different body sizes.

The explanation based on sexual selection has come to be accepted as the most plausible of these. Among mammals where SSD occurs, males are usually the larger sex—females are the larger sex only in Mysticeti, some phocids, spotted hyaenas, *Crocuta crocuta*, some Lagomorpha, and some Chiroptera (Ralls, 1977). Studies of a range of mammalian taxa posit sexual selection as the likely primary force explaining male‐biased SSD; however, the extent of SSD differs between taxa and with ecological circumstances. For instance, SSD is associated with polygyny for primates (Lindenfors & Tullberg, 1998; Weckerly, 1998), ungulates (Perez‐Barberia, Gordon, & Pagel, 2002), and pinnipeds (Lindenfors, Gittleman, & Jones, 2007). In ruminants, species with harem‐based mating systems are more dimorphic than those with territorial, polygynous, and monogamous mating systems (Weckerly, 1998). Soulsbury, Kervinen, and Lebigre (2014) observed that SSD based on sexual selection could account for variation in the reproductive success of male mammals, a measure of the degree of sexual selection. Similarly, the analysis of mammalian breeding systems by Lukas and Clutton‐Brock (2013) showed a clear link between mating system and SSD, where male‐biased SSD is more common in species where females exhibit solitary life histories compared with socially monogamous species.

The importance of sexual selection in maintaining male‐biased SSD has been implicated in explaining an allometric pattern known as “Rensch's rule.” This rule states that, within a lineage, SSD is positively correlated with mean body size (hyperallometry) in taxa where males are larger, and negatively correlated (hypoallometry) where females are larger (Rensch 1950 cited by Abouheif & Fairbairn, 1997). Although Rensch's rule holds for a variety of taxa (e.g., Abouheif & Fairbairn, 1997; Dale et al., 2007; Fairbairn, 1997; Sibly, Zuo, Kodric‐Brown, & Brown, 2012), it is by no means universal. For example, it consistently fails where females are the larger sex (Webb & Freckleton, 2007). Furthermore, although the relationship holds for the Class Mammalia as a whole (Lindenfors et al., 2007), when individual Families are considered, only primates (Lindenfors & Tullberg, 1998), bovids (antelopes), cervids (deer), and macropods (kangaroos) exhibit a significant Rensch allometry (Sibly et al., 2012). Where a positive male‐biased SSD allometry is observed, however, sexual selection does not provide a complete explanation—it is also necessary to explain what controls female size. In cases of selection for larger size in males, selection for larger females may also occur due to direct genetic linkage (Kemper, Visscher, & Goddard, 2012; Lande, 1980) and indirect effects (i.e., females need to be larger to be able to produce larger male offspring; Lindenfors, 2002). However, because fecundity decreases with increasing size among female mammal species (Lindenfors et al., 2007), there can be counter‐acting selection for small female size in species needing to maintain high fecundity, which can also lead to a positive Rensch effect within taxa.

Isaac (2005) commented that the dominance of the sexual selection hypothesis was likely to be simplistic—other possible factors influencing SSD and Rensch allometry include the importance of infanticide in reproductive strategy (e.g., Opie, Atkinson, Dunbar, & Shultz, 2014), and factors that shape social systems (Dale et al., 2007). Ralls (1977) speculated that the quality and dispersion of food resources could oppose polygyny by influencing the dispersion of females and therefore how individuals organize their intra‐ and intersexual territories. The analysis of Lukas and Clutton‐Brock (2013) provided some support for this; transition from the ancestral state to social monogamy was associated with lower population density of individuals (adjusting for body size). They speculated this occurred where species came to rely on resources with high nutritional value, but low abundance. Their analyses also suggested that this led to increased competition among females, and lower female population densities. In these circumstances, it might not be possible for a male to defend more than one female, and the benefits of larger male size would therefore tend to be lost (Lukas & Clutton‐Brock, 2013). These studies suggest that ecological factors can alter the intensity of sexual selection, although the extent to which this may occur remains relatively unknown.

The Mustelidae present an informative group for exploring how diet and social system interact with SSD to produce a Rensch trend. Body mass within the family varies more than 100‐fold, and SSD ranges from parity, to males being more than twice the size of females. Furthermore, mustelids exhibit a range of trophic dependencies and substantial variability in their social organization (Johnson, Macdonald, & Dickman, 2000). Across the Carnivora as a whole, there is no Rensch trend (Fairbairn, 1997). But the Mustelidae show a negative Rensch allometry with SSD greatest in the smaller species (Moors, 1980; Ralls & Harvey, 1985), which is inconsistent with existing theory. However, Abouhief and Fairbairn (1997) questioned the robustness of this effect, as it was not corrected for phylogenetic dependency.

We examine this allometry here, expanding the taxonomic range of our analyses to include the superfamily Musteloidea (comprising the families Mustelidae, Procyonidae, Mephitidae, and Ailuridae). We first test the null hypothesis of no allometric trend in dimorphism with body mass, correcting for phylogenetic dependencies (Harvey & Pagel, 1991). We then describe how dimorphism varies with body size, diet type, and social system, exploring hypotheses linking asymmetric rates of body‐size selection to sexual selection (e.g., Dale et al., 2007; Lindenfors, 2002). We also explore whether dimorphism is related to body shape, specifically to elongation, as well as to litter size. The smallest mustelids are both elongate and highly dimorphic (Powell, 1979). The elongate form of small species results in high rates of heat loss and increased thermoregulatory costs (Brown & Lasiewski, 1972), which can create considerable energetic stress. Smaller mustelids also produce larger litters, which places high energetic demands on gestation and lactation (Gittleman & Thompson, 1988), potentially constraining body size.

We find that when omnivory, insectivory, or the consumption of aquatic prey intersect with larger body sizes, these species have the capacity to tolerate reduced individual food security by sharing access to food types that occur in rich patches (Macdonald & Johnson, 2015; Newman, Zhou, Buesching, Kaneko, & Macdonald, 2011) that are spatially configured such that they are less defendable than are terrestrial vertebrate prey (Johnson et al., 2000). Consequently, there is a tendency for larger, less carnivorous species to exhibit a territorial and mating strategy where males may not attempt to defend access to multiple females, thus conferring no advantage to larger male size. Conversely, small musteloids are predominately obligate carnivores, for which the dispersion of food resources promotes defendable, intrasexual territories (Powell, 1979). This spatial arrangement results in polygynous mating systems, and males must compete for access to females, conferring a selective advantage to larger males.

## 2 Material and Methods

We applied analyses to those species of the Musteloidea (i.e., the Mustelidae, Procyonidae, Mephitidae, and Ailuridae; see Ralls & Harvey 1985) listed by Wilson and Mittermeier (2009). We obtained body mass data from Johnson et al. (2000), augmented with additional data from the literature. Phylogenetic relationships were taken from a recent consensus phylogeny of the Carnivora (Agnarsson et al., 2010); no phylogenetic data were available for eight species. Additionally, sex‐specific body mass data were unavailable for 23 less well‐known species, yielding a sample size of 54 musteloid species for which data for both male and female body masses and phylogeny were available (see Table S1 and Appendix S1).

For consistency with previous studies, we quantified SSD as the ratio of mean male to mean female mass (see Abouheif & Fairbairn, 1997). Following Fairbairn (1997), we regressed log_e_ female mass against log_e_ male mass. We tested our null hypothesis of no allometric trend using the slope of this regression; a slope of significantly <1.0 supports the presence of the negative allometry. We used a model 1 regression, predicting male mass using female mass. Model 1 and model 2 solutions converge as the correlation between male mass and female mass approaches 1.0 (Webb & Freckleton, 2007). The correlation for our 54 musteloid species was 0.984.

To explore hypotheses linking body‐size asymmetry patterns to sexual selection, we tested for relationships between SSD and the following:

1. Body elongation. Under our null hypothesis of no allometry in body shape, the expected value for the slope of body length versus mass (on a log_e_ scale) is 0.33 (Ralls & Harvey, 1985). To test for trends in elongation with SSD, we used an index of elongation based on that defined by Ralls and Harvey (1985): head body length divided by mass^0.33^ × 100.
2. Diet. Defined according to Gittleman (1985), where a species’ main food source constitutes at least 60% of the diet, with the categories (1) carnivorous consumption of terrestrial prey (henceforth “carnivorous”); (2) carnivorous consumption of aquatic prey (henceforth “piscivorous”); (3) omnivorous; (4) insectivorous; and (5) herbivorous/frugivorous (henceforth “herbivorous”). We note that because many of the Lutrinae consumed a piscivorous diet mixed with aquatic invertebrates, the “piscivorous” category also encompassed those eating crabs and crayfish—as highlighted by Powell (1979), the diet of some otters is dominated by ectothermic or invertebrate prey.
3. Social system. Defined according to Johnson et al. *(* 2000) as “solitary”; “pair‐living”; “variable groups” (species that are variable, ranging from solitary to living in groups across populations); and “group‐living.”
4. Litter size. Defined as the mean number of offspring produced per parturition.

As with body mass, data for these traits were obtained from Johnson et al. (2000) and augmented with values from the literature. Sources for these data are detailed in Appendix 1.

We used the R (CRAN) “*MCMCglmm”* package (Hadfield, 2010) to control for phylogenetic dependencies. The program derives parameter estimates using a Bayesian framework; uninformative, inverse gamma priors were applied, as in (Noonan, Newman, Buesching, & Macdonald, 2015). The number of model iterations, thinning interval, and burn‐in period were determined using diagnostic tests in the *R* package “*coda”* (Plummer, Best, Cowles, & Vine, 2006), and convergence was confirmed using the Geweke diagnostic (Gewke, 1992). MCMCglmm models were also used to test for differences in dimorphism among dietary and social‐class categories. We used DIC (deviance information criterion), an analog of AIC (Akaike information criterion), for comparing models with and without individual predictors; while unsettled, research into model selection methods for Bayesian mixed models suggests this is a promising option (Barnett, Koper, Dobson, Schmiegelow, & Manseau, 2010). For categorical predictors, use of the default pMCMC values is unsatisfactory, as the number of tests carried out is equal to one less than the number of levels of the predictor, and the choice of baseline level is often arbitrary.

## 3 Results

### 3.1 Allometry of SSD

**Figure 1:**
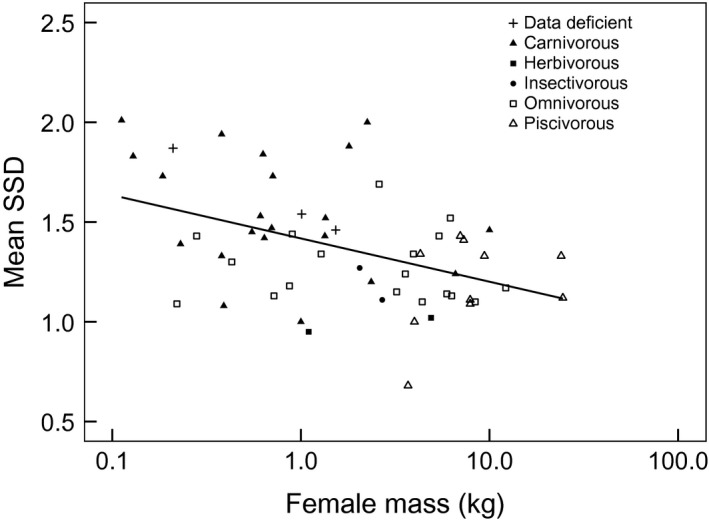
Scatter plot depicting the relationship between sexual size dimorphism (SSD) and female mass (kg) in musteloids, showing dietary classes. Data deficient species were those for which no accurate dietary data were available

Across the musteloids, SSD clearly decreased with body mass (Figure 1). The MCMCglmm Bayesian slope was 0.94 (95% CI: 0.88–0.99), and therefore significantly lower than 1.0. A similar pattern was evident when regressing the Mustelidae in isolation (slope = 0.92, 95% CI: 0.86–0.98). Interestingly, the negative allometry also existed among the nine *Mustela* spp. alone, where the confidence interval was significantly below 1.0 (slope = 0.71, 95% CI: 0.48–0.94). There were too few data for Mephitidae species for separate analysis, but these followed the same trend. Those Procyonidae for which we had data varied relatively little in both female mass and SSD, although again, small sample size precluded separate analysis.

### 3.2 SSD and elongation

There was no evidence for a trend in elongation with body mass across the Musteloidea, and the slope of the length mass relationship (log‐log scale) was consistent with 0.33 (slope = 0.30, 95% CI: 0.21–0.37). Nor was there any evidence for a trend in SSD with elongation index (slope = −0.085, 95% CI: −0.21–0.045, pMCMC = 0.25). A similar pattern was evident when regressing the Mustelidae in isolation (slope = −0.096, 95% CI: −0.23–0.058, pMCMC = 0.21). Again, there were too few data for Mephitidae and Procyonidae species for separate analyses, but these followed the same trend.

### 3.3 SSD and diet

**Figure 2:**
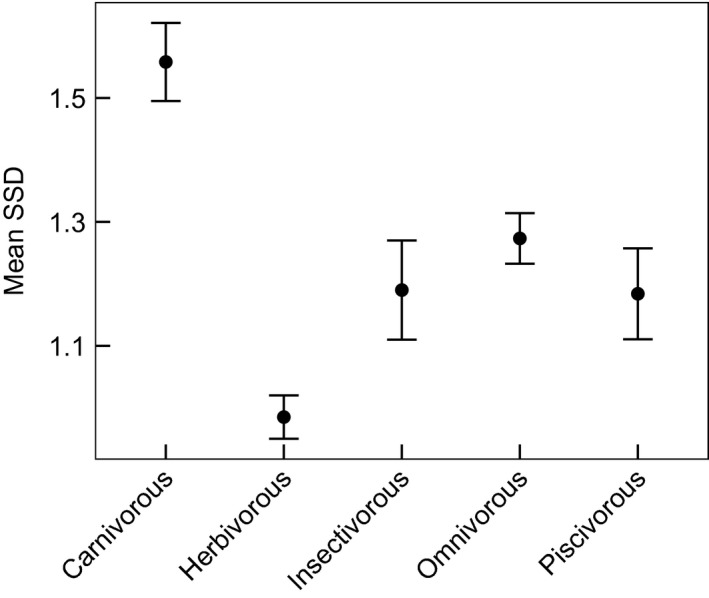
Mean dimorphism (±*SE*) in musteloids of each dietary class

Diet was related to log‐scaled mean adult mass. A model predicting mass using diet was superior to the intercept‐only model (ΔDIC = 5.5). Insectivores were significantly larger than carnivorous species (pMCMC = 0.03). Dietary class was also associated with SSD (Figure 2); highest SSD was observed among carnivorous musteloids, followed by piscivorous species, omnivores, insectivores, and herbivores. The model including diet was a better fit than the null model with intercept only (ΔDIC = 2.2), and SSD was significantly lower in omnivores, herbivores, and piscivores compared with carnivorous species (pMCMC = 0.05, 0.03, and 0.07, respectively).

### 3.4 SSD and social system

There was a significant relationship between social system and adult mass, where group‐living species were larger than solitary species (pMCMC = 0.0151). Mean dimorphism in solitary species was 1.38 (*SE* = 0.06 *n* = 32), which was higher than in group‐living species 1.23 (*SE* = 0.07, *n* = 14). But whereas the contrast was marginally significant when not adjusted for phylogeny (*F*_1,44_ = 3.53, *p* =0 .07), too few independent taxonomic clusters were present for this to be robust to phylogenetic correction (pMCMC = 0.44). Only two pair‐living species were identified, thereby preventing separate analyses.

### 3.5 Body mass and litter size

**Figure 3:**
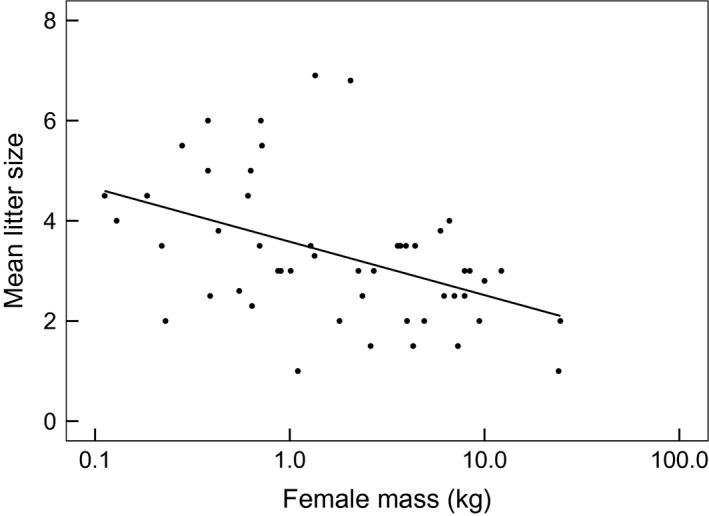
Scatter plot depicting the relationship between mean litter size and female mass (kg) in musteloids

Across the musteloids, litter size decreased with increasing female mass (slope = −0.11, 95% CI: −0.22–0.005, pMCMC = 0.069; Figure 3). The pattern among the Mustelidae alone was very similar (slope = −0.12, 95% CI: −0.23–0.003, pMCMC = 0.057). Again, there were too few data for Mephitidae and Procyonidae species, precluding separate analyses.

## 4 Discussion

In those taxa for which a positive Rensch effect has been reported, it is invariably associated with polygynous mating systems and assumed to be the product of sexual selection, operating through body‐size advantages. However, there is no consensus on the mechanisms involved in determining SSD in those taxa that counter the Rensch allometry. This suggests that “Rensch's rule,” and the primary importance of sexual selection generally (Lindenfors et al., 2007) provide an insufficient explanation for patterns of SSD.

We demonstrate a negative Rensch allometry in the Musteloidea, whereas previous observations combining all Carnivora together, or examining all nonmustelid carnivorans, have not shown any over‐arching effect (Abouheif & Fairbairn, 1997; Fairbairn, 1997). Rather than an explanation of SSD based on body size, our analyses pointed to a fundamental role of diet. SSD is highest among those musteloid species with a diet dominated by high energy, less abundant items (e.g., small vertebrate prey), and is lower where low energy, abundant items dominate the diet (e.g., for insectivory and mixed omnivory). We propose that although the present findings describe only the Musteloidea, the mechanisms involved are likely to be influential in determining socio‐spatial geometries and mating systems of mammals generally (Lukas & Clutton‐Brock, 2013; Macdonald & Johnson, 2015).

There are examples where the diet‐driven effect on SSD can be illustrated between closely related taxa. For instance, carnivorous *Spilogale putorius* (spotted skunk) exhibits greater SSD than omnivorous *Mephitis mephitis* (striped skunk). Similarly, omnivorous *Aonyx capensis* (African clawless otter) is less dimorphic than piscivorous *Lutrogale perspicillata* (smooth‐coated otter). Crucially, we show that SSD also varies with group‐living propensities (although the latter effect could not be disentangled from phylogenetic dependence), where the ability to form groups is the product of diet type, resource dispersion, and body size (Macdonald & Johnson, 2015). Here, for the musteloids, but also as a generalization warranting further exploration, we argue that the conventional explanation for the Rensch effect, exclusively invoking sexual selection, breaks down because feeding ecology interacts with the ability of males to compete for access to multiple females. That is, dietary diversity in this versatile superfamily (Macdonald & Johnson, 2015) results in a tendency for the advantage of male size to be obviated.

We propose that by influencing how females organize intrasexual territories (Johnson et al., 2000; Powell, 1979) and, consequently, how males optimize their access to mates, feeding ecology shapes reproductive strategies. Among musteloids, obligate carnivores are least able to share a minimum defendable territory, whereas, provided individuals are large enough to be able to tolerate the reduced food security sharing brings (Johnson et al., 2000; Macdonald & Johnson, 2015), omnivores are more able to share territories, and insectivores have the least exclusive territories (Johnson et al., 2000; Macdonald & Johnson, 2015); see also (Noonan et al., 2015). Musteloids living in pairs, or groups, tend to be among the least dimorphic species in the Superfamily. For example, in Britain, a rich patchy resource (earthworms) promotes shared territories and group‐living in *Meles meles* (European badgers; reviewed in Johnson, Jetz, & Macdonald, 2002). *M. meles* exhibits a SSD ratio of only 1.1, and Johnson and Macdonald (2001) speculated that a competitive advantage to large male size in *M. meles* was unlikely as females mate with multiple males (see also Dugdale, Macdonald, Pope, Johnson, & Burke, 2008) and males have little confidence in paternity (Macdonald & Johnson, 2015), which may further contribute to parity of the SSD ratio. Similarly, a dietary basis for group‐living has also been proposed for several otter species (Johnson et al., 2000). For instance, unpredictability and patchiness of fish distribution in ox‐bow lakes prevent group‐living giant otter, *Pteronura brasiliensis*, males from defending access to more than one breeding female (Groenendijk et al., 2015), and consequently, this species exhibits relatively low SSD.

Conversely, the spatial organization of carnivorous musteloids involves intrasexual territoriality. This is exacerbated among very small species (clustered in the upper left in Figure 1), which are vulnerable to conditions of low food security (Newman et al., 2011). In these species, the territories of males tend to be much larger than those of females, typically encompassing more than one female (Powell, 1979). We observe that solitary, territorial musteloids exhibited the greatest SSD within the Superfamily. The advantage of larger male size is enhanced in species where males provide little or no parental investment (Trivers, 1972), where freedom from postnatal involvement allows males to invest instead more time and energy into competing for mating opportunities as a means of increasing reproductive success. Interestingly, there are transitional species, exemplified by *Martes* spp., which, albeit being adaptable generalists, consume varied diets (Zhou et al., 2011) and exhibit male‐biased SSD. *Martes* spp. are unable to sustain groups because reduced secondary food security exceeds their threshold of tolerance (Newman et al., 2011) and males tend to exhibit a facultative polygynous mating system (Clutton‐Brock 1989). Furthermore, examining the mating strategy of *Martes foina*, Genovesi, and Boitani (1997) reported paternal‐investment polygyny, where the male continues his association with his offspring as they mature, although without direct paternal provisioning.

We also observed that litter size was greater among smaller, more dimorphic musteloids. However, although the relationship between female mass and litter size in the Musteloidea is consistent with patterns across the Mammalia generally (Healy et al. 2014), the positive relationship between SSD and litter size is counter to the negative relationship observed by Carranza (1996). It is well established that within taxa, fecundity is lower in larger bodied, K‐selected species, compared with smaller, r‐selected species (Allaine et al., 1987; Healy et al. 2014). At smaller sizes, the selection for energetic efficiency can be extreme in females rearing large litters. For instance, a female *Mustela erminea* (stoat), weighing 150 g, might give birth to a litter of up to 15 neonates, each weighing ca. 3 g (King, 1983). This 30‐g litter (discounting placental weight) would comprise 30% of her nonpregnant weight. In contrast, a female *M. meles,* weighing 12 kg, giving birth to a maximum of three cubs, each with a neonatal weight of 75 g, adds <2% of her mass (Lariviere & Jennings, 2009). Moors (1980), however, demonstrated the energetic advantage of smaller female size for *M. nivalis,* where a lactating female requires about 20% less energy (equivalent to 45–55 additional short‐tailed voles over the lactation period) than if it were as big as a male. Consequently, the male‐biased SSD in smaller musteloids is comprised of constraints on selection for larger female size (Lindenfors et al., 2007) interacting with sexual selection promoting larger size in males (resonating with the Moors–Erlinge hypothesis MEH; see Powell & Leonard, 1983). This selection for female “smallness,” particularly at the lower end of the mass spectrum where reproductive costs are more extreme provides a further explanation for the drivers behind the negative Rensch allometry described here. A corollary of the MEH is that, where food is abundant, males should realize a competitive advantage (Lindenfors et al., 2007), whereas constraints on female size persist. This is based on the rationale that body size is, to some extent, food limited (Mcnab, 1980) and when per capita food intake permits, larger males will be selected for. Exactly the expected pattern was observed when carnivorous *Pekania pennanti* (fishers) were introduced into a site in the USA with an abundant food supply unexploited by other predators—15 years postintroduction, males had undergone selection to be larger than individuals from the founder population, whereas females had not changed in size (Powell, 1979).

Powell (1979) also linked SSD in the Mustelidae to body shape, noting that the most dimorphic species were also more elongate and carnivorous. Elongation was attributed as an adaption for hunting in burrows (see also Martin, 1989; Noonan et al., 2015), a hunting mode which is particularly important for smaller females dependent on smaller prey (King, Powell, & Powell, 2007). Related to this, Gliwicz (1988) suggested that the body diameter of female mustelids might be limited so that when it increases due to pregnancy, they can still access the burrows of their prey. While small species that forage in burrows are also among the most elongate in the group, we found no general relationship between SSD and elongation in our much larger sample of species (regardless of phylogenetic correction).

In summary, while numerous taxa follow a positive Rensch allometry (Abouheif & Fairbairn, 1997; Dale et al., 2007; Fairbairn, 1997), the Musteloidea show the opposite trend, exhibiting greater SSD among smaller species. We attribute this at least partly to feeding ecology, in instances when omnivory and insectivory result in mating systems where defending access to multiple females is not a viable male strategy (Macdonald & Johnson, 2015; Noonan et al., 2015). The selective advantage of male size interacts with reduced fecundity (Healy et al. 2014) and reproductive efficiency (Lindenfors et al., 2007) associated with larger female size, imposing significant constraints on females. We conclude that diet and resource dispersion promote social and mating systems that undermine the advantage of large male size, by reducing the extent to which contest competition contributes to male reproductive success.

## Conflict of Interest

None declared.
