# Supplementary material for: Sexual size dimorphism in musteloids: An anomalous allometric pattern is explained by feeding ecology
Source: Ecol Evol. 2016 Oct 29;6(23):8495–501. doi: 10.1002/ece3.2480 (PMC5167046; doi:10.1002/ece3.2480)
Supplement: Supplementary file 1 [file ECE3-6-8495-s001.pdf]

Appendix S1

References for sources of Musteloidea data detailed in Table S1.

1.Anderson, E., Forrest, S.C., Clark, T.W. & Richardson, L. (1986). Paleobiology, biogeography, and systematics of the black-footed ferret, *Mustela nigripes* (Audubon and Bachman), 1851. *Great Basin Naturalist Memoirs*, 0, 11–62.

2.Balharry, E., Jefferies, D.J. & Birks, J.D.S. (2008). Pine marten. In: *Mammals of the British Isles: handbook* (eds. Harris, S. & Yalden, D.W.). Mammal Society, Southampton, pp. 447–455.

3.Begg, C., Begg, K. & Kingdon, J. (2013). *Mellivora capensis* Ratel (Honey Badger). In: *Mammals of Africa. V Carnivores, Pangolins, Equids and Rhinoceroses* (eds. Kingdon, J. & Hoffmann, M.). Bloomsbury Publishing, London, pp. 119–125.

4.Buskirk, S.W. & McDonald, L.L. (1989). Analysis of Variability in Home-Range Size of the American Marten. *The Journal of Wildlife Management*, 53, 997–1004.

5.Caryl, F.M. (2008). Pine marten diet and habitat use within a managed coniferous forest. *PhD Thesis*, University of Stirling.

6.Clark, T.W., Anderson, E., Douglas, C. & Strickland, M. (1987). *Martes americana. Mamm sp*, 289, 1–8.

7.Copeland, J.P. & Whitman, J.S. (2003). Wolverine. In: *Wild Mammals of North America Biology, management and conservation* (eds. Feldhammer, G.A. & Thompson, B.C.). Baltimore and London, pp. 672–682.

8.Cuzin, F. (2013). *Poecilictis libyca* Lybian Striped Weasel. In: *Mammals of Africa. V Carnivores, Pangolins, Equids and Rhinoceroses* (eds. Kingdon, J. & Hoffmann, M.). Bloomsbury Publishing, London, pp. 90–92.

9.d'Inzillo Carranza, I. & Rowe-Rowe, D.T. (2013). *Hydrictis maculicollis* Spotted-necked otter. In: *Mammals of Africa. V Carnivores, Pangolins, Equids and Rhinoceroses* (eds. Kingdon, J. & Hoffmann, M.). Bloomsbury Publishing, London.

10.de Villa-Meza, A., Avila-Flores, R., Cuarón, A.D. & Valenzuela-Galván, D. (2011). *Procyon pygmaeus* (Carnivora: Procyonidae). *Mamm sp*, 43, 87–93.

11.Dragoo, J.W. & Sheffield, S.R. (2009). *Conepatus leuconotus* (Carnivora: Mephitidae). *Mamm sp*, 827, 1–8.

12.Ford, L.S. & Hoffmann, R.S. (1988). *Potos flavus. Mamm sp*, 1–9.

13.Gao, Y.T. & Wang, S. (1987). *Fauna Sinica: Mammalia. Vol. 8. Carnivora.* Science Press, Beijing.

14.Gompper, M.E. (1996). Sociality and asociality in white-nosed coatis (*Nasua narica*): foraging costs and benefits. *Behavioral Ecology*, 7, 254–263.

15.Gompper, M.E. & Decker, D.M. (1998). *Nasua nasua. Mamm sp*, 580, 1–9.

16.Gorsuch, W.A. & Larivière, S. (2005). *Vormela peregusna. Mamm sp*, 779, 1–5.

17.Groenendijk, J. & Hajek, F. (2006). *Giants of the Madre de Dios.* Frankfurt Zoological Society, Lima.

18.Harris, S. & Yalden, D.W. (2008). *Mammals of the British Isles: handbook.* 4 edn. Mammal Society, Southampton.

19.Harrison, R.L. (2012). Ringtail (*Bassariscus astutus*) Ecology And Behavior In Central New Mexico, USA. *Western North American Naturalist*, 72, 495–506.

20.Heptner, V.G. & Sludskii, A.A. (2002). *Mammals of the Soviet Union. Vol. II, part 1b, Carnivores (Mustelidae and Procyonidae).* Smithsonian Institution Libraries and National Science Foundation, Washington D.C.
21.Hornocker, M.G. & Hash, H.S. (2011). Ecology of the wolverine in northwestern Montana. *Canadian Journal of Zoology*, 59, 1286–1301.
22.Hwang, Y.T. & Larivière, S. (2001). *Mephitis macroura. Mamm sp*, 686, 1–3.
23.Hwang, Y.T. & Larivière, S. (2005). *Lutrogale perspicillata. Mamm sp*, 786, 1–4.
24.Jones, K.L., Van Vuren, D.H. & Crooks, K.R. (2008). Sudden Increase in a Rare Endemic Carnivore: Ecology of the Island Spotted Skunk. *Journal of Mammalogy*, 89, 75–86.
25.Kays, R.W. (2000). The behavior and ecology of olingos (*Bassaricyon gabbii*) and their competition with kinkajous (*Potos flavus*) in central Panama : Mammalia. *Mammalia mamm*, 64.
26.King, C.M. & Powell, R.A. (2007). *The Natural History of Weasels and Stoats: Ecology, Behavior, and Management.* 2nd edn. Oxford University Press, Oxford.
27.Kinlaw, A. (1995). *Spilogale putorius. Mamm sp*, 511, 1–7.
28.Kruuk, H. (2006). *Otters: Ecology, behaviour and conservation.* Oxford University Press, Oxford.
29.Laidre, K.L., Estes, J.A., Tinker, M.T., Bodkin, J., Monson, D. & Schneider, K. (2006). Patterns of growth and body condition in sea otters from the Aleutian archipelago before and after the recent population decline. *Journal of Animal Ecology*, 75, 978–989.
30.Lanszki, J. & Valkár, B. (2009). Data for external morphometry of stone marten, polecat and weasel in Hungary. *Natura Somogyiensis*, 227–230.
31.Lariviére, S. (1998). *Lontra felina. Mamm sp*, 575, 1–5.
32.Lariviére, S. (1999). *Lontra longicaudis. Mamm sp*, 609, 1–5.
33.Lariviére, S. (2001). *Poecilogale albinucha. Mamm sp*, 681, 1–4.
34.Lariviére, S. (2002). *Ictonyx striatus. Mamm sp*, 698, 1–5.
35.Lariviére, S. & Lariviére, S. (2003). *Amblonyx cinereus. Mamm sp*, 720, 1–5.
36.Lariviére, S. & Walton, L.R. (1998). *Lontra canadensis. Mamm sp*, 1–8.
37.Lindzey, F.G. (2003). Badger *Taxidea taxus.* In: *Wild Mammals of North America Biology, Management, and Conservation* (eds. Feldhamer, G.A., Thompson, B.C. & Chapman, J.A.). London.
38.Lotze, J.-H. & Anderson, S. (1979). *Procyon lotor. Mamm sp*, 1–8.
39.Macdonald, D.W., Harrington, L.A., Yamaguchi, N., Thom, M.D. & Bagniewska, J.M. (2015a). Biology, ecology and reproduction of American mink, *Neovison vison*, on lowland farmland. In: *Farming and Wildlife. Conflict in the countryside* (eds. Macdonald, D.W. & Feber, R.E.). Oxford, pp. 126–147.
40.Macdonald, D.W., Newman, C. & Buesching, C.D. (2015b). Badgers in the rural landscape - conservation paragon or farmland pariah? Lessons from the Wytham Badger Project. In: *Wildlife Conservation on Farmland Volume II: Conflict in the Countryside* (eds. Macdonald, D.W. & Feber, R.E.). Oxford, pp. 65–94.
41.Magoun, A.J. (1985). Population characteristics, ecology, and management of wolverines in northwestern Alaska. *PhD Thesis*, Fairbanks.
42.Masuda, R. (2015). *Martes melampus* (Wagner, 1840). In: *The Wild Mammals of Japan* (eds. Ohdachi, S.D., Ishibashi, Y., Iwasa, M.A., Fukui, D. & Saitoh, T.). Kyoto, Japan.

43.McDonald, R.A. (2013). *Mustela nivalis* Least Weasel (Common Weasel)/ *Mustela subpalmata* Egyptian Weasel. In: *Mammals of Africa. V Carnivores, Pangolins, Equids and Rhinoceroses* (eds. Kingdon, J. & Hoffmann, M.). Bloomsbury Publishing, London, pp. 85–87.

44.McFadden, K.W. & Meiri, S. (2013). Dwarfism in insular carnivores: a case study of the pygmy raccoon. *Journal of Zoology*, 289, 213–221.

45.McFadden, K.W., Gompper, M.E., Valenzuela, D.G. & Morales, J.C. (2008). Evolutionary history of the critically endangered Cozumel dwarf carnivores inferred from mitochondrial DNA analyses. *Journal of Zoology*, 276, 176–186.

46.Medellín, R.A., Ceballos, G. & Zarza, H. (1998). *Spilogale pygmaea. Mamm sp*, 600, 1–3.

47.Monakhov, V.G. (2011). *Martes zibellina* (Carnivora: Mustelidae). *Mamm sp*, 43, 75–86.

48.Nowak, R.M. (2005). *Walker's Carnivores of the World.* 6 edn. John Hopkins University Press, Baltimore.

49.Ohdachi, S.D., Ishibashi, Y., Iwasa, M.A., Fukui, D. & Saitoh, T. (Eds.). (2015). *The Wild Mammals of Japan.* Shoukadoh Book Sellers and the Mammal Society of Japan.

50.Poglayen-Neuwall, I. & Toweill, D.E. (1988). *Bassariscus astutus. Mamm sp*, 327, 1–8.

51.Powell, R.A. (1981). *Martes pennanti. Mamm sp*, 156, 1–6.

52.Presley, S.J. (2000). *Eira barbara. Mamm sp*, 636, 1–6.

53.Reyes-Küppers, R. (2007). Ecology and Behaviour of the Southern River Otter *Lontra provocax* Thomas 1908 in Chile. *PhD Thesis*, Osnabrück.

54.Roberts, M.S. & Gittleman, J.L. (1984). *Ailurus fulgens. Mamm sp*, 222, 1–8.

55.Rosas, F.C.W., Rocha, C.S.D., Mattos, G.E. de & Lazzarini, S.M. (2009). Body weight-length relationships in giant otters (*Pteronura brasiliensis*) (Carnivora, Mustelidae). *Brazilian Archives of Biology and Technology*, 52, 587–591.

56.Sasaki, H., Ohta, K., Aoi, T., Watanabe, S., Hosoda, T., Suzuki, H., et al. (2014). Factors Affecting the Distribution of the Japanese Weasel *Mustela itatsi* and the Siberian Weasel *M. sibirica* in Japan. *Mammal Study*, 39, 133–139.

57.Sidorovich, V., Kruuk, H. & Macdonald, D.W. (1999). Body size, and interactions between European and American mink (*Mustela lutreola* and *M. vison*) in Eastern Europe. *Journal of Zoology*, 248, 521–527.

58.Somers, M.J. & Nel, J.A.J. (2013). *Aonyx capensis.* In: *Mammals of Africa. V Carnivores, Pangolins, Equids and Rhinoceroses* (eds. Kingdon, J. & Hoffmann, M.). Bloomsbury Publishing, London.

59.Stuart, C. & Stuart, T. (2013). *Poecilogale albinucha* African Striped Weasel. In: *Mammals of Africa. V Carnivores, Pangolins, Equids and Rhinoceroses* (eds. Kingdon, J. & Hoffmann, M.). Bloomsbury Publishing, London.

60.Valenzuela, D. (1998). Natural history of the white-nosed coati, *Nasua narica*, in a tropical dry forest of western mexico. *Revisita Mexicana de Masozoologica*, 3, 26–44.

61.Van Gelder, R.G. (1968). The genus Conepatus (Mammalia, Mustelidae): variation within a population. *American Museum novitates*, 2322, 1–37.

62.Verts, B.J. Carraway, L.N. (1998). *Land mammals of Oregon.* University of California Press, Berkeley.

63.Wang, Q. (1990). *The Mammal Fauna of Anhui.* Anhui Publishing, Hefei.

64.Weir, R.D. & Bio, R.P. (2003). Status of the fisher in British Columbia. *Conservation Data Centre, and B.C. Minist. Water, Land and Air Protection, Biodiversity Branch,*

Victoria, BC. Wildl. Bull., B-105, 1–38.
65. Wereszczuk, A. & Zalewski, A. (2015). Spatial Niche Segregation of Sympatric Stone Marten and Pine Marten–Avoidance of Competition or Selection of Optimal Habitat? *PLoS ONE*, 10, e0139852.
66. Yensen, E. & Tarifa, T. (2003a). *Galictis cuja. Mamm sp*, 728, 1–8.
67. Yensen, E. & Tarifa, T. (2003b). *Galictis vittata. Mamm sp*, 727, 1–8.
68. Youngman, P.M. (1990). *Mustela lutreola. Mamm sp*, 362, 1–3.
